# Comparison of the clinical performance of self-adhering flowable composite and resin-based pit and fissure sealant: a randomized clinical trial in pediatric patients

**DOI:** 10.1186/s12903-024-04449-6

**Published:** 2024-08-14

**Authors:** Elham Farokh Gisour, Fatemeh Jahanimoghadam, Reyhaneh Aftabi

**Affiliations:** 1 Social Determinants on Oral Health Research Center, Kerman University of Medical Sciences, Kerman, Iran; 2 Endodontology Research Center, Kerman University of Medical Sciences, Kerman, Iran

**Keywords:** Pit and fissure sealants, Self-adhering flowable composite, Retention, Marginal integrity

## Abstract

**Background:**

Self-adhering flowable composites are an innovative step in reducing the chair time of pit and fissure sealant treatment. This study aimed to compare the clinical performance of self-adhering flowable composite (SELF-ADH) and resin-based pit and fissure sealant (RBs).

**Methods:**

This is a double-blinded, randomized, split-mouth clinical trial conducted on 80 fully erupted permanent mandibular first molars from 40 children aged 6 to 12 years. For each participant, two permanent molars were randomly treated with SELF-ADH or RB. All sealants were assessed at 3, 6, and 12-month follow-ups considering retention, marginal integrity, marginal discolouration, colour matching, surface texture, and caries recurrence. To analyse the data, logistic regression and Fisher’s exact tests were used (significance level *P* < 0.05).

**Results:**

At the 12-month follow-up, the number of fully retained sealants in the SELF-ADH group was significantly higher than that in the RB group (*P* < 0.001). Also, The success rate of marginal integrity in the SELF-ADH group was significantly greater than the the RB group (*P =* 0.031), while the rate of sealant marginal discolouration was higher in the RB group (*P <* 0.001). The incidence of recurrent caries in teeth with partial loss of sealant in both groups (*P =* 0.004, *P <* 0.001) increased significantly over time.

**Conclusion:**

The retention and marginal integrity of the self-adhering flowable composite were significantly greater than those of the resin-based sealant. Therefore, due to the small number of work steps, flowable self-adhering composites can be used as alternatives to resin-based pit and fissure sealants.

**Trial registration:**

This study was approved by the Ethics Committee of Kerman University of Medical Sciences with the code IR.KMU.REC.1399.556 and Iraninan Registry of Clinical Trials (IRCT) code IRCT20180521039763N4, as well as full compliance with the Declaration of Helsinki.

## Introduction

Anatomical pits and fissures in teeth are the most common areas of incipient lesions and directly depend on the shape and depth of the pits and fissures, whereas lesions rarely develop on smooth surfaces that are easily cleaned [1]. The high efficiency and safety of pit and fissure sealants justify their common use as preventive agents [2]. Many clinical trials have revealed that the use of flowable composites instead of unfilled resin sealants could significantly increase sealant retention [1, 3, 4]. An innovative step forward in the field of flowable composites is the introduction of self-adhering flowable composites [4]. Self-adhering flowable composites (SAFCs) undergo etching, bonding and priming simultaneously; therefore, they are also called 8th-generation adhesive materials because they provide a link between 7th-generation adhesives (all in one) and flowable composites [5]. Self-adhesive composites have fewer application steps, a lower possibility of error, and, as a result, the shortest treatment time. This approach can be helpful when dealing with children and makes quadrant dentistry possible [6].

Vertise™ Flow is the first self-adhering flowable composite from the Kerr Company; this composite uses OptiBond technology and has an acidity of ∼ 1.9. This SAFC uses the functional monomer glycerophosphate dimethacrylate (GPDM) to condition the enamel and dentin simultaneously and a hydrophilic monomer (hydroxyethyl methacrylate) to enhance dentin bonding by increasing surface wetting and resin penetration, which allows dual bonding, chemical bonding between the functional monomer and hydroxyapatite of the tooth structure and micromechanical bonding, as the polymerized resin impregnates the collagen fibres and dentin smear layer. GPDM is a functional monomer with two polymerizable groups that can react with monomers in both bonding systems and resin composites, improving the quality of the polymer network and the physical properties of the hybrid layer [7].

The use of SAFCs is very cost-effective for the treatment of young children because pediatric dentists are always looking for restorative materials that reduce the time required for treatment, which is a critical factor in child cooperation [8]. Although the Vertise™ Flow composite has been under investigation in vitro and in vivo for almost 8 years, few clinical studies have been conducted regarding its success rate [8].

Considering the high prevalence of pit and fissure carious lesions, pit and fissure sealant treatment is a cost-effective preventive strategy. By reducing the number of clinical steps, self-adhering flowable composites promise the shortest treatment time for pediatric patients [9]. Owing to the paucity of clinical studies conducted on Vertise Flow composites (Kerr, Orange, CA, USA), this study focused on the clinical performance of resin-based pit, fissure sealants and self-adhering flowable composites.

## Method

### Ethical consideration

The present study is a randomized, double-blinded, split-mouth clinical trial conducted on children aged 6 to 12 years who were brought to the Pediatric Dentistry Department of Kerman University of Medical Sciences. This study was approved by the Ethics Committee of Kerman University of Medical Sciences with the code IR.KMU.REC.1399.556 in 21/1/2021 and IRCT code IRCT20180521039763N4 in 15/4/2021 (available at https://irct.behdasht.gov.ir/trial/54418), as well as full compliance with the Declaration of Helsinki. The study design followed the CONSORT 2010 Statement: Updated guidelines for reporting parallel group randomized trials (Schulz et al. 2010).

### Inclusion and exclusion criteria

The study included children with caries-free fully erupted mandibular permanent molar teeth with retentive pits and fissures and the possibility of cotton roll isolation. It should be considered that the two first molars which were chosen for each patient have to be on opposite sides. Uncooperative children with any history of systemic problems or allergies, parafunctional activity, use of any drugs affecting saliva flow or developmental disorders in the teeth were excluded from the study.

### Sample size calculation, allocation and randomization

**Fig. 1:**
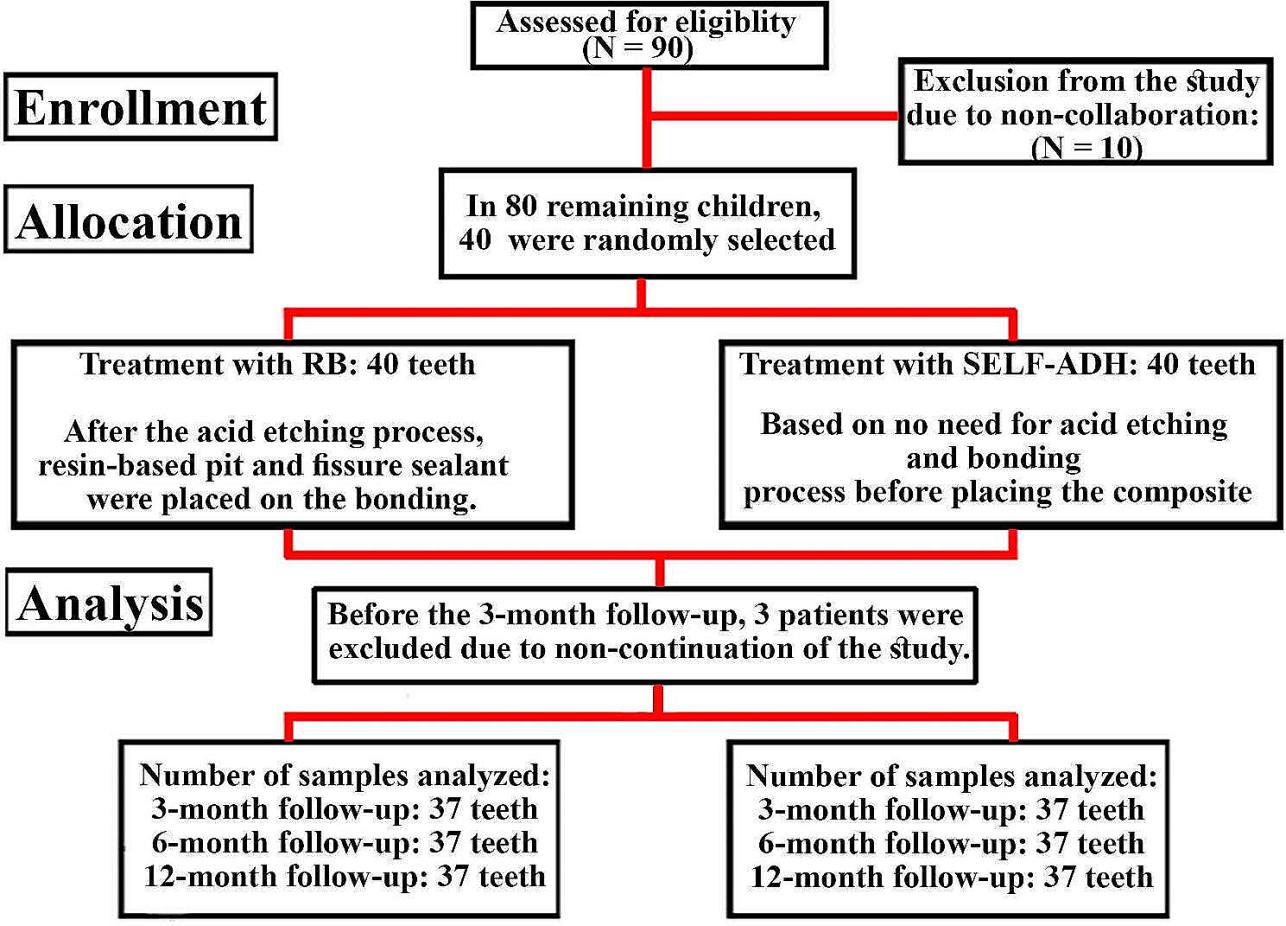
Consort flowchart SELF-ADH: self-adhering flowable composite, RB: resin-based pit and fissure sealant

The sample size (*n* = 30 teeth in each group) was calculated using G*Power software with a power of 80% at a 5% level of significance. With regard to possible loss to follow-up, the final total sample size was 80 teeth (40 teeth in each group). A total of 90 children were screened, out of whom only 10 were excluded because they did not meet the inclusion criteria. Randomization was performed with random-numbered tables, and 40 children were selected for the study (Fig. 1). A pediatric dentistry resident explained the objectives of the study to the parents. Participants were subsequently provided with oral health education and diet recommendations. Informed consent was subsequently obtained, and two bitewing radiographs were taken from the right and left molar areas to diagnose incipient interproximal lesions via the parallel technique and Kodak E-speed #2 film (Eastman-Kodak Co., Rochester, NY, USA), an RVG Sensor Holder, Trident, Italy) and a Planmeca Intra X-ray radiography unit (Helsinki, Finland). For patients who had recently undergone a radiograph, that radiograph was used.

Eighty studied teeth were divided into two groups of 40 examinees each: group 1, self-adhering flowable composite; and group 2, resin-based pit and fissure sealant. All teeth were treated by the same dentist (pediatric dentistry resident). We used a split-mouth design to eliminate the impact of confounding factors such as masticatory forces, site-specific oral hygiene behavior and dietray habits. For each child, the coin toss method was used to select the type of sealant to be applied first. In this way, each side of the coin was arbitrarily assigned to one of the two materials, and the type of material that should be placed on the teeth was determined by coin toss. The sealant was subsequently applied to the patient’s permanent first molar on the right or left side according to the lottery method; i.e., the words “right” and “left” were written on two sheets of paper, and then each was placed inside an envelope that was randomly opened for each patient to specify the side to which the material was previously determined.

### Clinical procedure

First, the surface of the teeth was cleaned using nonfluoridated prophylaxis paste, and then each tooth was isolated by placing cotton rolls in the buccal and lingual vestibules and using a high-volume saliva ejector. After that, the tooth surfaces of both groups were prepared for the placement of the materials. A short diamond fissurotomy bur (Diamond, Conical, 852, DIATESSIN, Switzerland) was used to open the fissures to the size of the bur tip; then, in each patient, the permanent first molar was covered by a self-adhering flowable composite (Vertise Flow, Kerr, Orange, CA, USA) with an A2 colour, and the permanent mandibular first molar on the other side was covered with a light cure resin pit and fissure sealant with an A2 colour (Master-Dent®, Dentonics, USA).

In group 1, following the manufacturer’s instructions for the self-adhering flowable composite (Vertise Flow), there was no need for acid etching or bonding before placing the composite. After washing and drying the tooth with compressed air, the self-adhering composite (with a maximum thickness of 0.5 mm) was placed on the occlusal surface using a microbrush for 20 s and cured for 40 s using a light cure unit (Coltene AG, Altstatten, Coltolux Switzerland). Finally, occlusion was evaluated using articulating paper.

In group 2, the occlusal surfaces of the molars were etched with 37% phosphoric acid gel for 20 s (Condac 37, FGM, Brazil), washed with water and dried using clean compressed air. In case of contamination of the etching surface with saliva, the tooth surface was etched again for 10 s. Then, two layers of Tetric N-Bond bonding agent (Ivoclar Vivadent, Switzerland) were added, a gentle flow of air was used for 5 s to evaporate the solvent, curing was performed for 20 s with a light curing unit, and the pit and resin fissure sealant (Master-Dent®, Dentonics, USA) were placed on the bonding agent. After ensuring that there were no bubbles, the sealant was cured for 40 s by slowly moving the probe on the pit and fissures. Finally, occlusion was evaluated.

### Calibration

Follow-up examinations were conducted at 3, 6, and 12 months. To confirm the intraexaminer reliability, before starting the follow-up evaluations, 10 teeth (10% of the total sample size) were selected from each group and examined by the operator and two pediatric dentistry specialists; however, no significant difference was observed in the examination data, and the kappa statistic for intraexaminer reliability was 0.85. The sealants were checked by a dental sickle double-headed probe (Dental Devices, Pakistan) and an intraoral mirror by the same operator and by two other operators if necessary. The retention rate, marginal integrity, marginal discolouration, colour match, surface texture of the sealants and presence of recurrent caries were evaluated using the Feigal criteria, Ryge criteria, and the modified version of the colour, coverage, caries (CCC) sealant evaluation system. We used these 3 criteria to evaluate all the clinical parameters related to sealant clinical performance. Additionally, these 3 criteria were chosen due to their simplicity, precise evaluation and good reliability [10–12].

At the one-year follow-up, to diagnose secondary caries caused by the sealant, a bitewing radiograph, which had the same characteristics as before, was obtained, and partial retained sealants were resealed.

### Blinding

In this double-blinded study, the participants and data analysts were kept blind to the groups; however, due to the difference between material colour and texture, it was not possible to keep the operator and outcome assessor blind.

### Statistical analysis

The data were analysed with SPSS version 26 statistical software, after which logistic regression and Fisher’s exact tests were used to analyse the data via the GEE method, and *P* values less than 0.05 were considered to indicate statistical significance. Also, Fisher’s exact test was used to compare the main clinical parameters (retention, marginal integrity and marginal discoloration) between the two groups of self-adhering composite and resin-based sealant. It should be noted that we used the multiple logistic regression test for analyzing the effect of demographic variables (age, gender) and also the role of the sealant material in relation to the dependent variables (retention, marginal integrity and marginal discoloration) in both groups. The GEE method helped us in order to analyse the multivariate longitudinal data with more accuracy.

## Results

### Demographic variables

Among the 80 examined teeth, among 14 girls and 23 boys, 6 were excluded because they did not complete the study, and the study included 74 teeth. The average age of the girls in the study was 7.3 (± 3.06) years, and that of the boys was 9.4 (± 1.9). Moreover, there was no significant difference between the two groups (*P =* 0.065).

### Retention rate

**Fig. 2:**
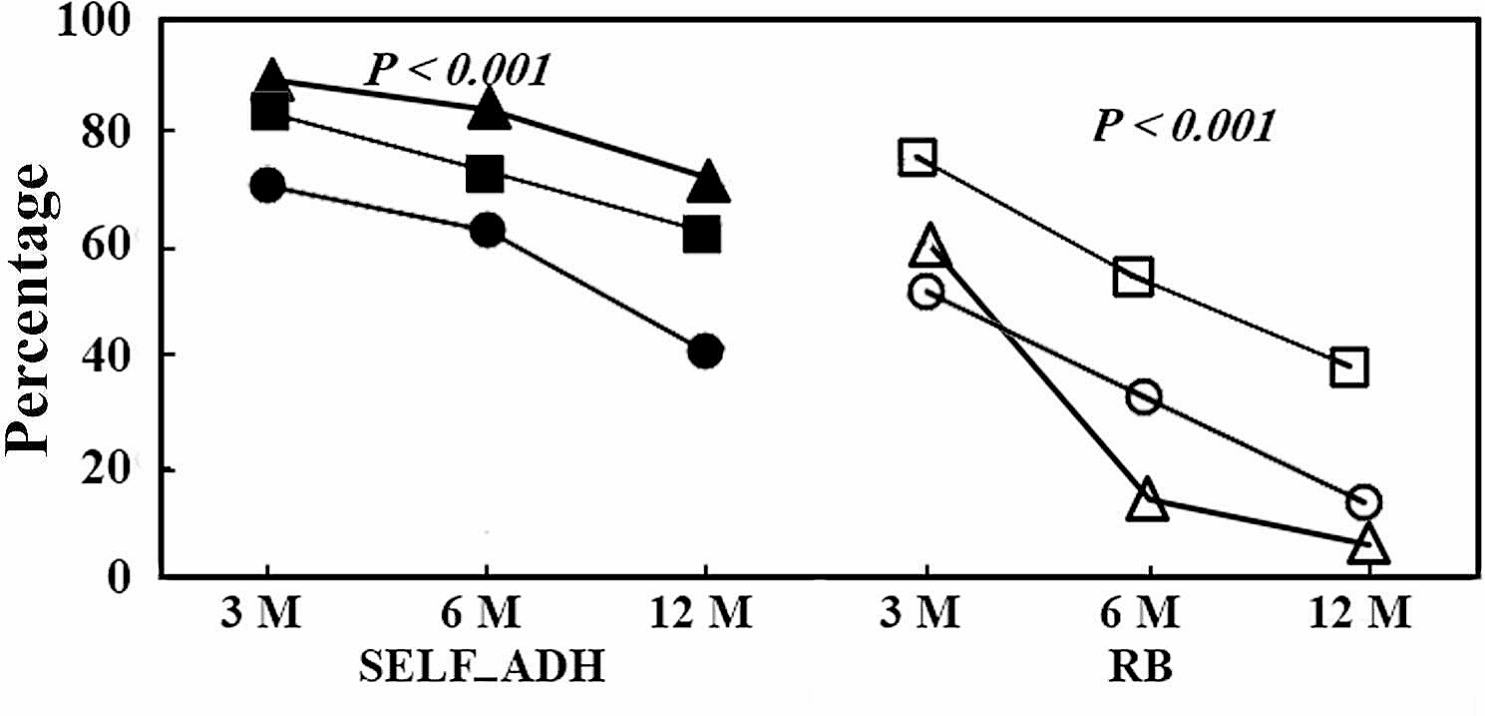
Comparison between SELF-ADH and RB in terms of retention, marginal integrity and marginal discolouration. The symbols for SELF-ADH are as follows: marginal discolration (solid triangle), marginal integrity (solid square) and retention (solid circle). The symbols for RB are marginal integrity (square), retention (circle) and marginal discolouration (triangle)

After 3 months, the total retention rate of the sealant was significantly greater in the SELF-ADH group than in the RB group (*P =* 0.001). At the 6-month follow-up, the superiority of the SELF-ADH group over the RB group was significant (*P =* 0.002). At the one-year follow-up, in the SELF-ADH group, 40.5% of the sealants had total retention, and 59.5% had partial retention. However, in the RB treatment, 13.5% of the genes had complete retention, and 86.5% had partial retention (*P =* 0.001) (Table 1). The retention rate of the sealant in both groups decreased significantly over time, although the decrease in the RB group was significantly greater (Fig. 2).

With regard to the time dependence of the observations, logistic regression revealed that after one year, the total chance of retention in the SELF-ADH group was 5.37 times greater than that in the RB group (*P <* 0.001). With increasing age, the total chance of retention also increased (*P =* 0.024). However, sex did not affect retention (*P =* 0.480).

**Table 1:** Retention rate according to evaluation time and group based on the Feigal criteria

Evaluation time (month)	Groups	Total retention	Relative retention	Total los	*P* value*
		Frequency (%)	Frequency (%)	Frequency (%)	
3	SELF-ADH	26 (70.3)	11 (29.7)	0 (0.0)	< 0.001*
	RB	19 (51.4)	18 (48.6)	0 (0.0)	
6	SELF-ADH	23 (62.2)	14 (37.8)	0 (0.0)	0.002*
	RB	12 (32.4)	25 (67.6)	0 (0.0)	
12	SELF-ADH	15 (40.5)	22 (59.5)	0 (0.0)	0.001*
	RB	5 (13.5)	32 (86.5)	0 (0.0)	

*: Fisher’s exact test

### Marginal integrity

The success rates of the marginal integrity of the sealant at the 3- and 6-month follow-ups in the SELF-ADH group were 83.8% and 73%, respectively, which were higher than the success rates of the same variable in the RB group at the same time, even though the difference was not significant (*P =* 0.073 and *P =* 0.282). Nevertheless, at the 12-month follow-up, the success rate of marginal integrity in the SELF-ADH group was significantly greater than that in the RB group (*P =* 0.031) (Table 2).

The marginal integrity rate of the sealant decreased over time in both groups, and the rate of decrease was significantly greater in the RB group (Fig. 2).

The results of the logistic model showed that the chance of success in the SELF-ADH group was 2.31 times greater than that in the RB group (*P =* 0.013), and the chance of success was 3.06 times greater in the girls group than in the boys group (*P =* 0.029); however, age had no significant effect on the marginal integrity of the sealant (*P =* 0.468).

**Table 2:** The success rate of sealant marginal integrity according to the evaluation time and group based on the Feigal criteria

Evaluation time (month)	Groups	Failure (%)	Success (%)	*P* value*
3	SELF-ADH	6 (16.20)	31 (83.80)	0.282
	RB	9 (2.30)	28 (75.70)	
6	SELF-ADH	10 (27)	27 (73)	0.073
	RB	17 (45.90)	20 (54.10)	
12	SELF-ADH	14 (37.80)	23 (62.20)	0.031*
	RB	23 (62.20)	14 (37.80)	

*: Fisher’s exact test

### Marginal discolouration

Compared with that in the RB group, the marginal discolouration in the SELF-ADH group was significantly lower at the 3- and 6-month follow-ups (*P =* 0.003 and *P <* 0.001). At the 1-year follow-up, the marginal discolouration in the SELF-ADH and RB groups was 28.6% and 91.8%, respectively, which was significantly greater in the RB group (*P* < 0.001). Additionally, in the SELF-ADH group, the success rate was greater than that in the RB group at all times (*P <* 0.05); i.e., the amount of marginal discolouration in the SELF-ADH group was significantly lower at all times (Table 3). As shown in Fig. 2, the marginal discolouration of the sealant increased over time in both groups, and the increase was significantly greater in the RB group.

**Table 3:** The success rate of sealant marginal discolouration according to the evaluation time and group based on the Feigal criteria

Evaluation time (month)	Groups	Failure (%)	Success (%)	*P* value*
3	SELF-ADH	4 (10.80)	33 (89.20)	0.003*
	RB	15 (40.50)	22 (59.50)	
6	SELF-ADH	6 (16.20)	31 (83.80)	< 0.001*
	RB	32 (86.40)	5 (13.60)	
12	SELF-ADH	12 (28.60)	25 (71.40)	< 0.001*
	RB	34 (91.80)	3 (8.10)	

*: Fisher’s exact test

### The color matching and surface texture rate

The color matching percentages of sealants in the SELF-ADH subgroup at the 3- and 6-month follow-ups were 97.3% and 89.2%, respectively, while in the RB group, the percentages were 100% and 91.9%, respectively, at the 3- and 6-month follow-ups. Therefore, throughout the 6-month follow-up, the RB group was slightly better than the SELF-ADH group was in terms of color matching with the tooth, although the difference was not significant. At the 1-year follow-up, color matching was high in both groups, and no significant difference was observed between them. The variable of colour matching in SELF-ADH and RB sealants showed an increase in score over time (*P =* 0.010 and *P =* 0.022).

The surface texture variable in SELF-ADH did not change substantially over time, and all the sealants had the same surface texture as tooth enamel. In RBs at the 3-, 6-, and 12-month follow-ups, only one sealant showed a difference in surface texture; however, it was not porous, and therefore, the surface texture changes in the RB group were not significant (*P =* 0.368).

The incidence rate of **secondary** caries.

In the SELF-ADH group, no incipient enamel caries were observed at the 3- or 6-month follow-up, while in the RB group, there was one cavitated enamel caries lesion at the 3- or 6-month follow-up; however, the difference between the two groups was not significant (*P* = 0.890). At the 12-month follow-up, in the SELF-ADH group, one cavitated enamel caries lesion was observed, and six sealants with partial retention also showed yellow‒brown discolouration at the base of pits and fissures, where the sealant was lost. At the 12-month follow-up in the RB group, no new cavitated enamel lesions had formed, but in seven partial recurrent sealants, yellow‒brown discolouration was observed. In this regard, the difference between the two groups at the one-year follow-up was not significant, and the probability of caries recurrence in teeth with partial loss of sealant increased significantly over time in both the SELF-ADH (*P =* 0.004) and RB (*P <* 0.001) groups.

## Discussion

Fissure sealant is implemented as part of community-based preventive oral health programs that have been effective for more than two decades [3, 13].

The present study showed that the retention rate at 3, 6, and 12 months was significantly greater for Vertise Flow (SELF-ADH) than for Master-Dent (RB).

Bhuvaneswari et al. (2022) indicated that self-adhering flowable composites (Dyad Flow, Kerr, USA) had significantly greater retention than unfilled sealant after multiple months of follow-up, which confirms the results of our study [14].

Additionally, Bagherian and Shirazi (2018) reported that the retention rate of flowable composites as sealants was better than that of RBs [4]. Another similar study by Wadhwa et al. (2018) also indicated that the retention rate of self-adhering flowable composite (Dyad Flow) was significantly greater than that of RB (Helioseal-F); however, all the findings are consistent with the present study [15].

However, Singh et al. (2019) did not observe a significant difference between the retention of flowable composites and conventional resin-based sealants in children aged 6–9 years [16]. Additionally, Jafarzadeh et al. (2010) suggested that despite the higher retention of flowable composites, there was no significant difference between them. Interestingly, the difference between these results and the present study could be due to the different types of sealant and flowable composite used and possibly the age range of the participants [17].

Additionally, Kucukyilmaz and Savas (2015) reported that the retention of self-adhering flowable composites (vertise flow) was significantly lower than that of flowable composites (tetric EvoFlow). In contrast, the retention of the highly filled pit and fissure sealant (Fissurit FX) and highly filled nanohybrid pit and fissure sealant (Grandio Seal) were also slightly greater than that of Vertise Flow, which is not consistent with the present study and can be related to the use of highly filled sealants [18].

ElEmbaby and El Tantawi (2019) showed that the retention rate of self-adhering flowable composites (Fusio liquid Dentin) did not significantly decrease over time. The inconsistency with our study may be related to the difference in the type of self-adhering composite used [19].

Among the reasons for the higher retention of self-adhering composites, we can point out the ease of use, high flow, fewer air bubbles, and increased working time [13]. Additionally, the decreased polymerization shrinkage reduced microleakage and improved retention. On average, approximately 5% of the volume of flowable composites undergoes polymerization shrinkage [20]. In this regard, Kusai Baroudi et al. concluded that the filler fraction of flowable composites has a great effect on their polymerization shrinkage strain [20]. In addition, the abrasion resistance of flowable composites is improved due to the presence of a higher filler content, and as a result, the possibility of partial or total loss is reduced [21]. These factors can contribute to the higher retention of flowable composites compared to sealants.

Regarding the success of a sealant, marginal integrity is always as important as retention. In the present study, the success rates of the marginal integrity of the sealant in the SELF-ADH (vertise flow) group were 83.8%, 73%, and 62.2% at 3, 6, and 12 months, respectively. In the RB group, 75.7%, 54.1%, and 37.8% of sealants were affected, respectively, and these percentages were greater than those in the RB group at all follow-up times, especially at 12 months. Wadhwa et al. reported (2018) [15] that the marginal integrity rate of self-adhering flowable composite (Dyad flow) as a sealant was better than that of RB (Helioseal-F), which further supported our findings. In a study by ElEmbaby et al. (2019), the marginal integrity of the self-adhering flowable composite (Fusio liquid Dentin) was high at the 6-, 12-, and 24-month follow-ups, which is consistent with the results of the present study [19]. In a study by Oz et al. [22], the marginal integrity of the self-adhering flowable composite (vertise flow) significantly decreased at the 12-month follow-up. Our study showed that these changes in the marginal integrity of the self-adhering composite are statistically significant over time.

Since the SAFC bonding material and the composite are cured simultaneously, the polymerization shrinkage cannot have much effect on the bonding strength. This feature increases the marginal integrity of self-adhering composites [23].

In the present study, the marginal discolouration rate of the sealant in both groups increased significantly over time, while at all follow-up times, the discolouration rate in the SELF-ADH group was lower than that in the RB group. This finding is similar to the results of ElEmbaby and Oz, where the self-adhering flowable composite always underwent significant marginal discolouration over time. However, these studies did not compare this variable with resin-based sealants [19, 22, 23]. The results of our study regarding the increase in the marginal discolouration of vertise flow over time confirmed the results of Sabbagh et al. [8].

It is well known that marginal integrity and marginal discolouration are related to the sealing ability of the sealant material and its ability to reduce microleakage. The general consensus is that self-etch adhesives and composites cannot form resin microtags in the enamel or create an acceptable hybrid layer; as a result, microleakage occurs over time [5]. However, Vichi et al. (2013) showed that self-adhering composites have significantly less microleakage than conventional flowable composites. This is possibly related to the fact that self-adhering composites have greater water absorption, which causes more hygroscopic expansion, reduces microleakage and ultimately produces a better seal [5]. The results of the present study also showed that Vertise Flow was significantly more successful than resin sealant in terms of both marginal integrity and marginal discolouration.

The color-matching changes in the present study were significant in both groups, but at the one-year follow-up, the changes were considered acceptable in both groups; *these findings are similar to those of ElEmbaby et al.* [19] but disagree with the results of Oz et al. [22], who reported that the surface discolouration of the Vertise Flow was not significant at the 5-year follow-up. This may be related to the variance of the studied groups. In the present study, the surface texture variable did not significantly change over time in any of the groups, whereas ElEmbaby et al. [19] suggested that surface texture changes were significant over time. This difference may be related to the difference in the SAFC and the presence of fusio liquid dentin. Oz et al. [22]. showed that Vertise Flow completely maintained its surface texture over a period of 5 years, which confirms the results of the present study.

Although caries prevention is the main goal of fissure sealant treatment [24], in the SELF-ADH (vertise flow) group, enamel lesions did not occur at the 3- or 6-month follow-up, and caries were observed in only 2.70% of the samples at the last follow-up. In the RB group, one of the sealants had enamel lesions with partial retention at the 3-month follow-up. This damage was immediately restored, and the lesion was considered enamel after the subsequent follow-up. Therefore, the incidence of enamel lesions at the end of 12 months in the second group was also 2.70%, and no significant difference between the groups was evident. Despite this, the possibility of caries recurrence in partially retained sealants in both groups increased significantly over time. Our interpretations agree with Bagheri et al. (2022) that there was no significant difference in terms of caries recurrence between filled and unfilled sealants [24]. This finding also confirms the findings of ElEmbaby et al. [19], who showed the absence of secondary carious lesions in teeth treated with self-adhering flowable composites.

In our study, a fissureotomy was performed to prepare pits and fissures, which removed plaque and debris and roughened the tooth enamel surface, thereby increasing the bond strength. This is a very important step in increasing the bond strength of self-adhering composites with the enamel surface, as confirmed by Wadhwa et al. (2018) [15]. Because the self-etching acidic primer in SAFCs has a lower acidity than phosphoric acid, this gap in enamel bonding needs to be compensated by fissureotomy [15]. In contrast to our study, Bhuvaneswari et al. [14]. and Eliades et al. [25]. used selective etching instead of fissurotmy to increase the bond strength. However, the retention of self-adhering and unfilled sealant in both studies did not differ from that in the present study.

The results of various studies did not reveal a significant difference between isolation by cotton rolls or rubber dams regarding sealant retention. On the other hand, due to the absence of local anaesthetic injection in the treatment of pit and fissure sealants, cotton rolls are always preferred in this clinical situation [19]. In the present study, the highest rate of total sealant retention loss occurred during the first 6 months, possibly due to differences in the cooperation ability of the participants, nutritional patterns, knowledge of oral health and personal hygiene habits, which were also suggested by Dhar et al. [26]. However, Bhuvaneswari et al. [14]. reported that the maximum sealant loss in self-adhering flowable composites (Dyad Flow, Kerr, USA) and unfilled resin sealants occurred between 6 and 12 months after treatment, which is inconsistent with our study. This difference may be related to the use of different types of self-adhering flowable composites and unfilled sealants.

In this study, the total sealant retention increased significantly in both groups as children aged, possibly because older children had a greater level of coping ability and cooperation in the dental environment.

In pediatric dentistry, the degree of cooperation of the child plays a key role in choosing the type of restoration. The success of composite restoration depends largely on the technique used and proper isolation. In uncooperative children, moisture control is not possible, so the success rate of restoration is low [27]; thus, in the present study, children with Frankel cooperation levels 3 or 4 were included.

Notably, because of the promising results of this study and because flowable composites can easily penetrate shallow or wide fissures [17], the use of self-adhering flowable composites (vertise flow) as fissure sealant materials seems to be reasonably efficient. Additionally, the use of these composites for the treatment of young children, especially precooperative patients, is very cost effective because pediatric dentists are always looking for restorative materials with high retention that reduce the time required for treatment [20].

Due to the limitation of sample size, it was not possible to consider permanent maxillary first molar teeth or compare the performance of SELF-ADH with that of conventional flowable composites and glass ionomers.

In future clinical research, longer follow-up times, larger sample sizes, more teeth, and new tooth preparation methods, particularly lasers, air abrasion, air polishing, etc., should be considered for further studies.

## Conclusion

A self-adhering flowable composite (vertise flow) as a pit and fissure sealant showed a significantly greater retention rate after one year of follow-up than did RB (master-Dent), although both experienced a significant decrease in retention over time.

The marginal integrity of the self-adhering flowable composite was greater than that of the RB composite, although both materials decreased significantly over time, and the reduction rate in the RB treatment was greater.

Both materials had high caries prevention properties over the course of one year, but there was no significant difference in the caries recurrence rate between the two materials.

The efficiency and success rate of the self-adhering flowable composite were greater for all the variables. Compared to RBs, due to the reduction in the number of steps and treatment time, RBs can be a suitable alternative to conventional sealants in clinical situations.

Also, it is recommended that future studies in this area, consider the longer follow up times for determining the retention of the novel composite material over greater periods of time. We suggest that future studies focus should remain on the main interfering factors that affect sealants longevity and durability. We hope that the promising novel self-adhering flowable composite shines bright in the treatment of uncooperative pediatric patients.

## Data Availability

The datasets generated and/or analysed during the current study are not publicly available due to all contributors specific reasons. For furthur information, please contact the corresponding author: Reyhaneh Aftabi, Email: reyhane9495@gmail.com.
